# Parkes Weber syndrome, a rare case of pulmonary hypertension: a case report

**DOI:** 10.1093/ehjcr/ytaf381

**Published:** 2025-08-20

**Authors:** Camila Castillo-Tello, Clemente Barron-Magdaleno, Consuelo Orihuela-Sandoval, Eduardo Gutiérrez-León

**Affiliations:** Department of Cardiology, National Institute of Medical Sciences and Nutrition Salvador Zubiran, Vasco de Quiroga 15, Belisario Domínguez Secc 16, Tlalpan, Mexico City 14080, Mexico; Faculty of Medicine, Universidad Nacional Autónoma de México, Circuito Interior, Avenida Universidad 3000, Ciudad Universitaria, Coyoacán, Mexico City 04510, Mexico; Heart Failure Clinic, Department of Cardiology, National Institute of Medical Sciences and Nutrition Salvador Zubiran, Vasco de Quiroga 15, Belisario Domínguez Secc 16, Tlalpan, Mexico City 14080, Mexico; Heart Failure Clinic, Department of Cardiology, National Institute of Medical Sciences and Nutrition Salvador Zubiran, Vasco de Quiroga 15, Belisario Domínguez Secc 16, Tlalpan, Mexico City 14080, Mexico; Heart Failure Clinic, Department of Cardiology, National Institute of Medical Sciences and Nutrition Salvador Zubiran, Vasco de Quiroga 15, Belisario Domínguez Secc 16, Tlalpan, Mexico City 14080, Mexico; Echocardiogram Subdivision, Department of Cardiology, National Institute of Medical Sciences and Nutrition Salvador Zubiran, Vasco de Quiroga 15, Belisario Domínguez Secc 16, Tlalpan, Mexico City 14080, Mexico; Division of Postgraduate Studies, Faculty of Medicine, Universidad Nacional Autónoma de México, Circuito de Posgrados s/n, Ciudad Universitaria,Coyoacán, Mexico City 04510, Mexico; Department of Medical Education, National Institute of Medical Sciences and Nutrition Salvador Zubirán, Vasco de Quiroga 15, Belisario Domínguez Secc. 16, Tlalpan, Mexico City 14080, Mexico

**Keywords:** Parkes Weber syndrome, Arteriovenous malformation, Pulmonary hypertension, Case report, Amputation, Heart failure

## Abstract

**Background:**

Parkes Weber syndrome (PWS) is a rare congenital vascular syndrome characterized by complex capillary malformation , venous malformation, lymphatic malformation, and arteriovenous malformation (AVM) in the affected limb with overgrowth; the latter is a pathognomonic feature that differentiates it from Klippel–Trenaunay syndrome. Cardiovascular complications include increased cardiac output, which promotes the onset of heart failure and the development of pulmonary hypertension (PAH), significantly impairing the quality of life due to severe functional class deterioration. However, these complications are currently treatable by ligation or removal of malformations.

**Case report:**

A 33-year-old male with a long-standing, progressively enlarging AVM of the right upper limb presented with necrosis and haemorrhage, leading to hypovolemic shock. Angiography revealed an AVM involving the subclavian, axillary, and brachial arteries, necessitating embolization and surgical interventions. Six years later, he developed distal finger necrosis, requiring infracondylar amputation. He later presented with stump infection, purulent discharge, orthopnoea, jugular venous distension, a loud second heart sound, and a holosystolic murmur in the tricuspid region. Echocardiography and catheterization confirmed PAH and a high-flow arteriovenous fistula. Multidisciplinary evaluation led to definitive amputation and PAH treatment. Postoperatively, PAH resolved, and the patient was discharged with ongoing follow-up, showing significant improvement.

**Discussion:**

This case report highlights the importance of a multidisciplinary approach in managing PWS, especially when endovascular interventions are not feasible owing to the diffuse nature of the AVM. It also emphasizes the potential for reversing severe complications through definitive surgical intervention in complex cases of PWS.

Learning points

- Consider the impact that arteriovenous malformations may have on the overload of the heart chambers, resulting in heart failure and pulmonary hypertension (PAH), which, together with the manifestations of Parkes Weber Syndrome, contribute to a poor prognosis for the patient if not addressed in a timely and appropriate manner by a multidisciplinary team.
- Managing arteriovenous malformations through embolization may serve as an early measure to prevent cardiovascular complications.
- Amputation of the affected limb was considered a definitive treatment for PAH secondary to the high output of the arteriovenous fistula.

## Introduction

Parkes Weber syndrome (PWS) is a rare congenital vascular syndrome characterized by complex capillary malformation (CM), venous malformation, lymphatic malformation, and arteriovenous malformation (AVM) in the affected limb with overgrowth.^1^ Cardiovascular complications include increased cardiac output, which promotes the onset of heart failure (HF) and the development of pulmonary hypertension (PAH), significantly impairing the quality of life due to severe functional class deterioration. However, these complications are currently treatable by ligation or removal of malformations.^1^

In this case report, we describe the short- and long-term outcomes of a patient with PWS who presented with ischaemia and PAH due to an AVM in the subclavian artery and vein region. The condition was treated with amputation of the affected limb, resulting in immediate correction of PAH.

## Summary Figure

**Table:** 

Date	Event
1996	Painful and pulsating mass, 3 cm in diameter
2008	Progression of the tumour with proximal extension involving the right forearm, superficial ulcerations with a tendency to bleed, oedema, purplish discolouration, and pain.
2010	Hypovolaemic shock cause by haemorrhagic, arteriovenous fistula repair and laser sclerotherapy
2016	necrosis affecting the second through fifth digits of the right hand Angiography: arteriovenous malformation in the right arm Investigation for RASA 1 mutation.
2022 October	Stump exhibiting signs of infection, high-output heart failure, pulmonary hypertension
2022 November	Remodelling of the stump, with no signs of pulmonary hypertension.

## Case report

**Figure 1:**
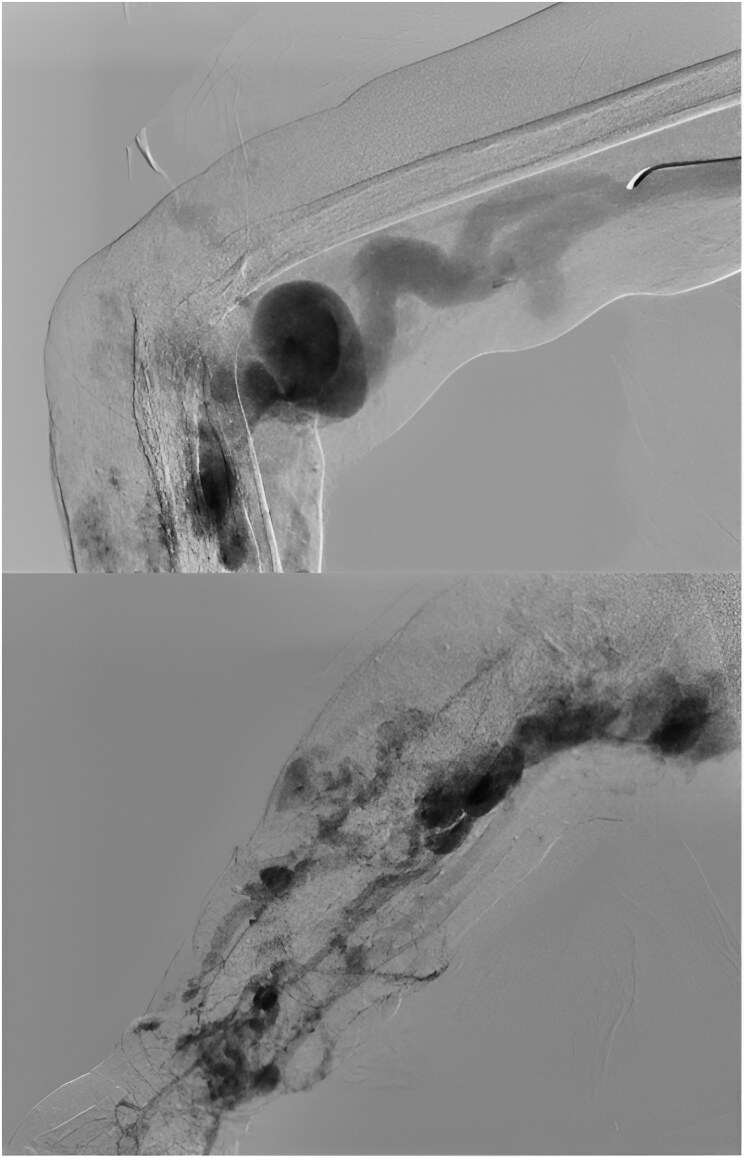
Showing diffuse active high-flow arteriovenous malformations originating from the subclavian, axillary, brachial, radial, and cubital arteries.

A 33-year-old male patient was referred to the hospital for the first time because of pain and oedema in the right hand, which had been evolving for 2 weeks. Upon examination, necrosis with visible bone was noted in the index, middle, ring, and little fingers of the right hand. The patient reported the appearance of a 3 cm pulsatile, painful, reddish-purple mass that was initially diagnosed as a haemangioma. However, over 14 years, the mass progressively enlarged, eventually affecting the right forearm, with the development of superficial ulcerations prone to bleeding, oedema, and pain. Two years later, the patient experienced hypovolemic shock secondary to haemorrhage from the mass, which required fluid resuscitation and haemostasis through suturing. For the first time, an angiography was performed, revealing an AVM dependent on the subclavian, axillary, brachial, radial, and ulnar arteries, as well as a high-flow fistula in the distal third of the forearm (*Figure 1*). The patient underwent embolization of the AVMs, endovascular ablation, surgical ligation, debridement, dressing changes, and skin grafting, resulting in improvement in both angiographic parameters, remission of the AVM, and clinical improvement of the affected limb.

**Figure 2:**
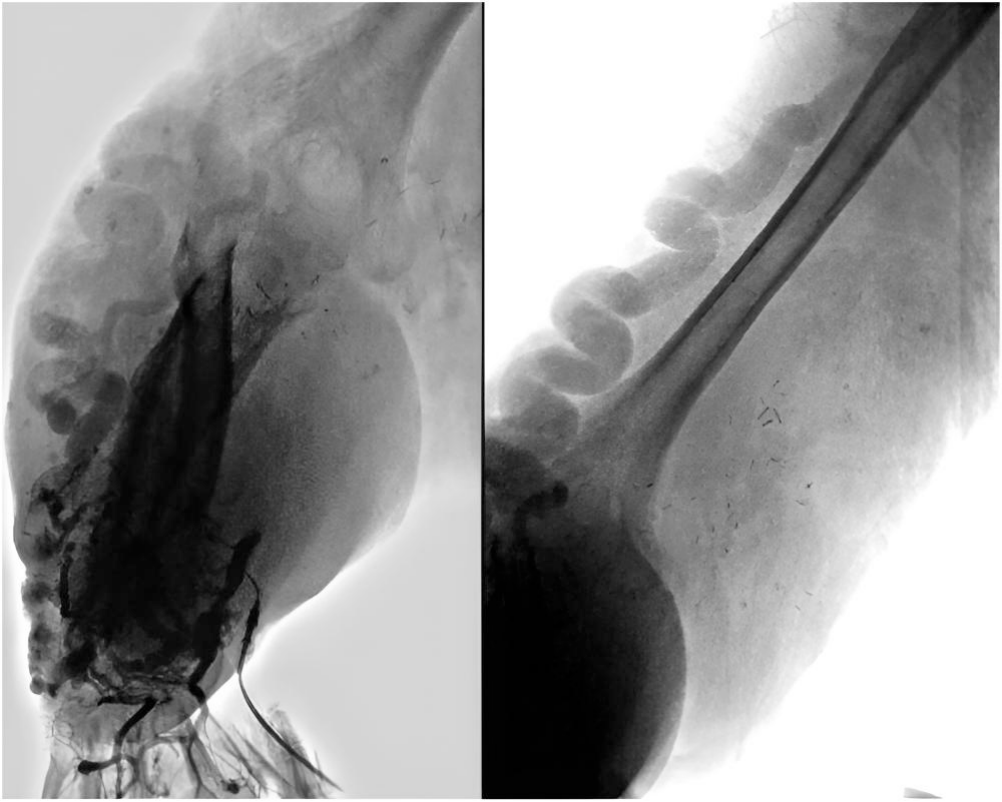
Arteriovenous malformation (AVM) in the right arm with arterial flow steal to the distal portion of the arm and hand.

Six years later, the patient was referred to our institute because of distal necrosis of the fingers 2–5. Angiography revealed an AVM involving the distal third of the brachial artery (*Figure 2*), leading to the recommendation of infracondylar amputation due to the persistence of the AVM.

During hospitalization, targeted genetic analysis for RASA1 mutations was negative. Notably, germline-inactivating RASA1 mutations are found in only 50–85% of PWS cases.^2^ Despite this, the diagnosis was confirmed based on clinical and imaging findings.

During follow-up, the patient developed an infection at the stump site, presenting with foul-smelling discharge, pain, warmth, oedema, fever, and night sweats, along with dyspnoea that progressed from exertion to rest, as well as palpitations. On clinical examination, hypertrophy of the right arm was noted, with multiple superficial varicosities and ulcers on the stump, exuding mucopurulent discharge with associated bleeding. There was orthopnoea, jugular venous distension grade III/IV, and auscultation revealed a loud second heart sound, a holosystolic murmur in the tricuspid region that increased with deep inspiration, blood pressure of 180/90 mmHg, and a heart rate of 120 b.p.m.

**Figure 3:**
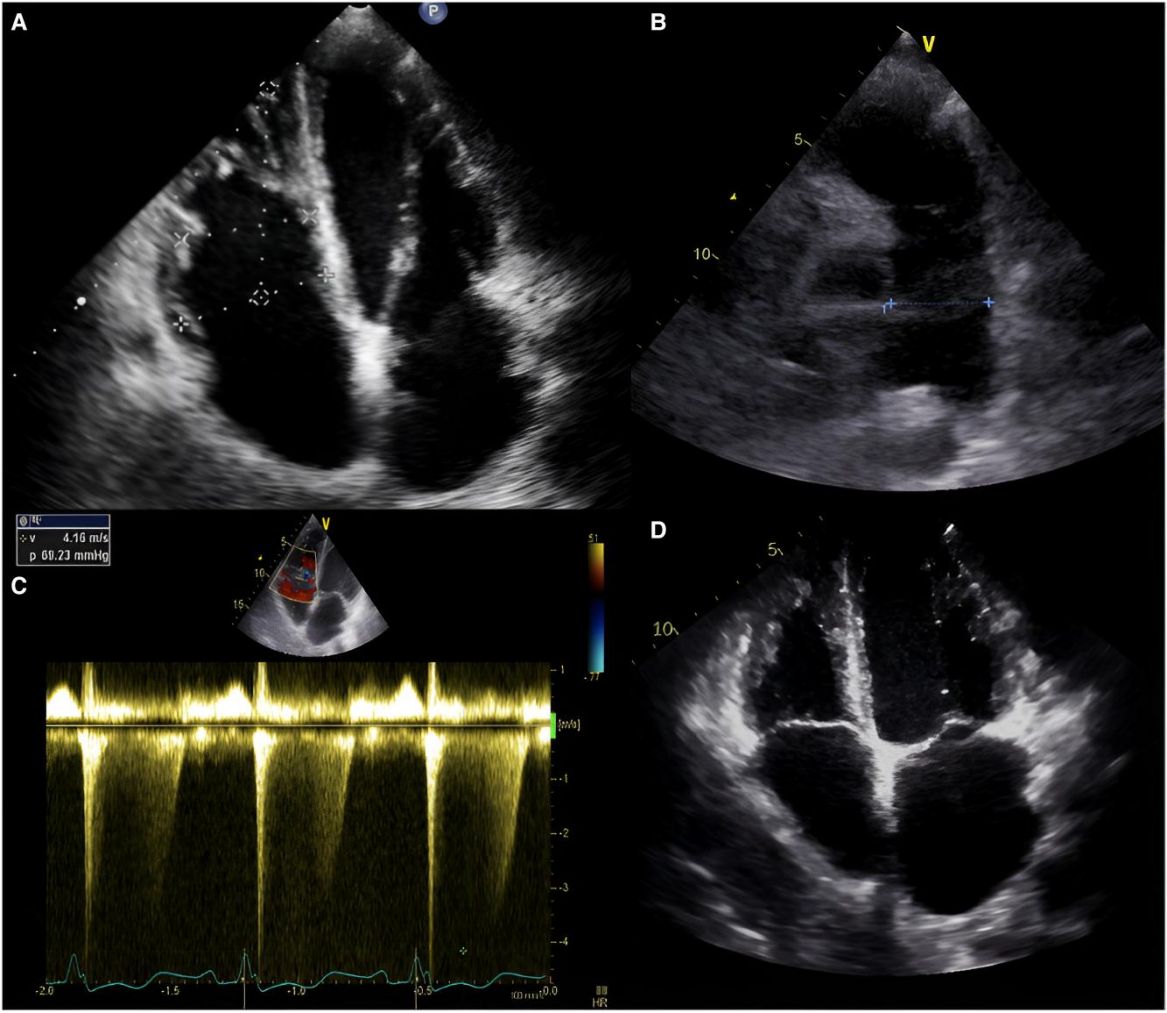
Transthoracic echocardiogram before and after amputation. (*A*) Four-chamber view showing dilation of the right ventricle and atrium with septal displacement. (*B*) Short-axis view demonstrating dilation of the pulmonary artery trunk at 48 mm. (*C*) Maximum tricuspid regurgitation velocity (TRV) of 3.73 m/s with a PAP of 69 mmHg. (*D*) Improvement in the diameters and function of the right chambers.

**Figure 4:**
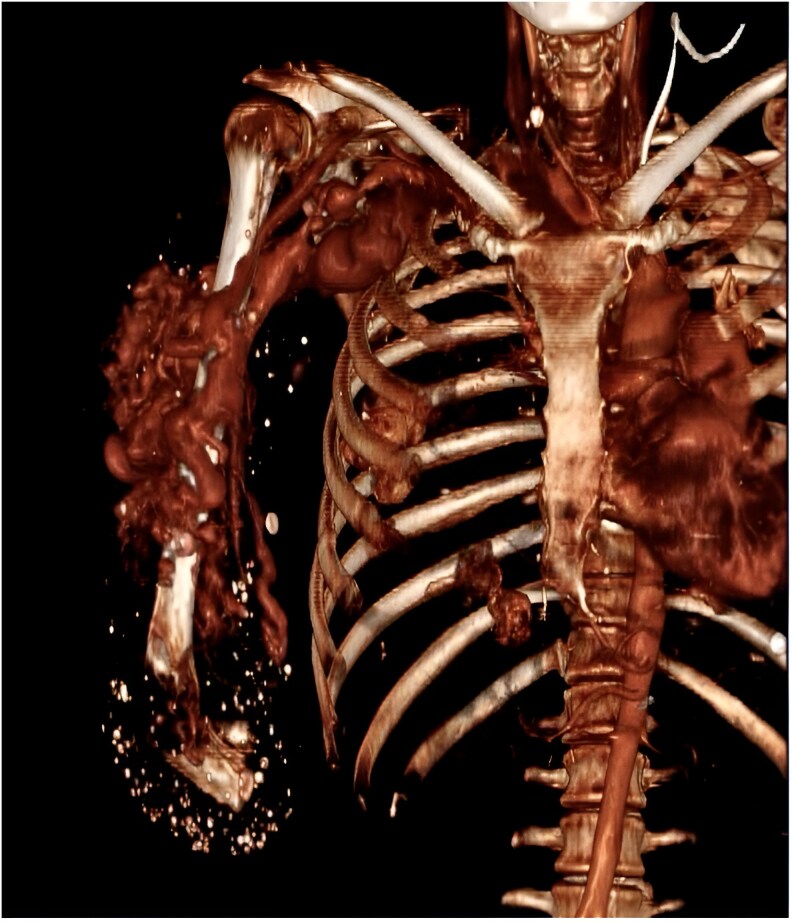
Patent arteriovenous fistula arising from the proximal third of the subclavian artery and vein.

**Figure 5:**
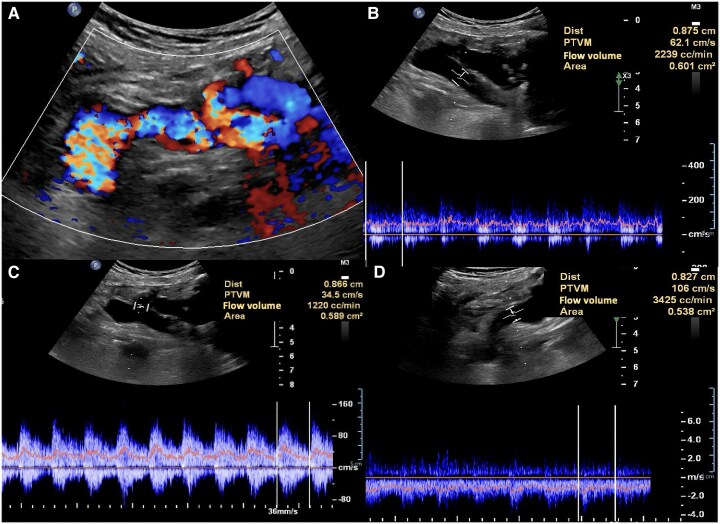
Patent arteriovenous fistula. (*A*) Turbulent flow, (*B*) spectral analysis demonstrates a flow volume of 2239 mL/min in the proximal third of the subclavian vein, (*C*) 1220 mL/min within the fistula, (*D*) 3425 mL/min in the subclavian artery distal to the fistula.

Chest radiography revealed cardiomegaly, primarily affecting the right chambers, a prominent pulmonary artery silhouette, and cephalization of flow, suggestive of PAH. Echocardiography showed chamber dilation (*Figure 3A*) without evidence of valvular abnormalities, preserved ejection fraction, a pulmonary artery diameter of 4.8 cm (*Figure 3B*), an estimated systolic pulmonary arterial pressure of 69 mmHg, and a maximum tricuspid regurgitation velocity (TRV) of 3.73 m/s (*Figure 3C*), indicating a high probability of PAH. The diagnosis was confirmed via right heart catheterization, which reported a mean pulmonary pressure of 40 mmHg and a pulmonary vascular resistance of 3.5 UW, total pulmonary resistances 3.9 UW transpulmonary gradient: 33 mmHg. A computed tomography angiography of the right upper limb revealed multiple tortuous and dilated vascular pathways, predominantly affecting the humeral and subclavian vessels, along with an arteriovenous fistula originating from the proximal third of the subclavian artery and vein (*Figure 4*). Doppler ultrasound showed a total flow rate of 5 L/min in the fistula (*Figure 5*).

A multidisciplinary evaluation concluded that amputation represented the most suitable option to enhance the patient’s quality of life. Simultaneously, treatment for PAH was commenced, and the management of HF was optimized using SGLT2 inhibitors, diuretics, spironolactone, and metoprolol. The procedure was successfully performed without complications. A follow-up echocardiogram demonstrated significant improvement in parameters, absence of chamber dilation, a pulmonary artery diameter of 2.2 cm, normal pulmonary flow (type 1), and normal TRV (*Figure 3D*), confirming the resolution of PAH. The patient was discharged with continued treatment and multidisciplinary follow-up. To date, the patient remains under observation.

## Discussion

Parkes Weber syndrome is characterized by extensive segmental CM, typically localized to a limb. The pathophysiological evolution of AVMs can be explained by the local mechanical compression of surrounding structures and the haemodynamic impact of arteriovenous shunting on circulation.^1,3^

There is no robust literature regarding the best therapeutic options, a systematic review available that includes 48 cases, reported that 12.5% of patients present with upper limb involvement due to AVMs; up to 50% of these cases are associated with high-output HF, and no cases of associated PAH were reported.^3^ Although there is no definitive treatment for vascular malformations, key management goals in PWS focus on improving the quality of life, slowing disease progression through excision or occlusion of existing AVMs, and preventing the development of HF and PAH.^3^ Although the most used therapeutic approach according to the literature is embolization followed by surgical amputation; amputation is also a recognized option in the management of HF associated with AVMs.^3^

Unfortunately, repeated embolization would not have been appropriate in this case, as prior attempts failed due to the diffuse nature of the AVM with multiple nidus points. Thus, the patient underwent a right arm amputation. In our case, the decision was made by a multidisciplinary team in line with literature reports that amputation is indicated when there is distal arterial ischaemia, a non-functional limb, severe HF, uncontrolled bleeding, or primary carcinoma.^4^

Our case differs from previously reported cases in that typically affects the lower extremities, whereas in this case, the upper limb was involved.^1^ This is a key factor in the rapid development of high-output HF and PAH, which is a rare finding. However, this allowed us to demonstrate the reversibility of these conditions in PWS following amputation of the affected limb; notably, this is the first case report to document such a therapeutic effect on PAH in a patient with PWS, highlighting it as a therapeutic option that should be further evaluated in future research and considered in complex cases that do not respond to initial measures.

This case report highlights the importance of a multidisciplinary approach, especially when endovascular interventions are not feasible owing to the diffuse nature of the AVM.

## Conclusion

A multidisciplinary approach is essential for managing PWS. Endovascular interventions have become a cornerstone in addressing AVM progression at an early stage; however, amputation remains crucial, particularly in patients with severe complications. Pulmonary hypertension is highly prevalent across numerous conditions, significantly limiting the quality of life and life expectancy. As in this case, management requires addressing the underlying pathology and understanding its origin for an effective treatment.

## Lead author biography


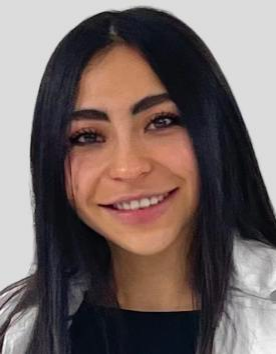


Camila Castillo-Tello received her medical degree from the National Autonomous University of Mexico, Mexico, with an interest in internal medicine and clinical cardiology.

## Data Availability

The data underlying this article will be shared on reasonable request to the corresponding author.
